# Supplementary material for: FDA-Approved Drug Screening for Compounds That Facilitate Hematopoietic Stem and Progenitor Cells (HSPCs) Expansion in Zebrafish
Source: Cells. 2021 Aug 20;10(8):2149. doi: 10.3390/cells10082149 (PMC8393331; doi:10.3390/cells10082149)
Supplement: Supplementary file 1 [file cells-10-02149-s001.zip › cells-1306180-supplementary.pdf]

Supplementary Material

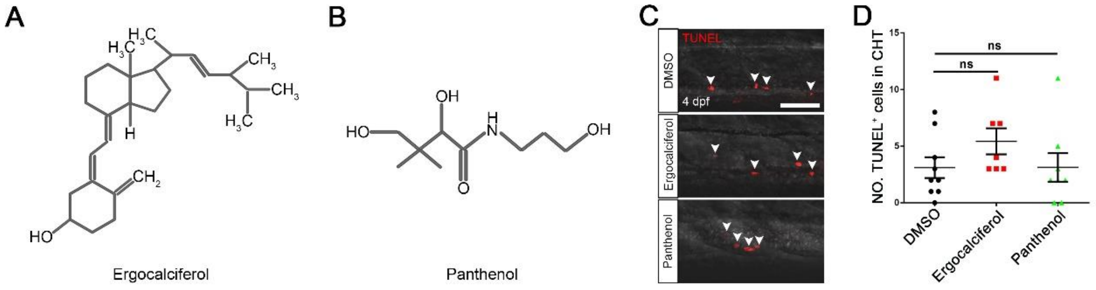

Figure S1: The structural formulas of ergocalciferol and panthenol and their effects on cell apoptosis. **(A-B)** The structural formula of ergocalciferol (A) and panthenol (B). **(C)** Confocal images of TUNEL assay after treating with either ergocalciferol or panthenol. White arrowheads indicate $TUNEL^+$ signals. **(D)** Statistical result of (C) (Mean ± SEM; DMSO, 3 ± 1, *n*=9; Ergocalciferol, 5 ± 1, *n*=7; Panthenol, 3 ± 1, *n*=8). Scale bar, 50 μm; ns, no significance.
